# Advancing *Strongyloides* omics data: bridging the gap with *Caenorhabditis elegans*

**DOI:** 10.1098/rstb.2022.0437

**Published:** 2024-01-15

**Authors:** Reem Al-Jawabreh, Dominika Lastik, Darrin McKenzie, Kieran Reynolds, Mona Suleiman, Angela Mousley, Louise Atkinson, Vicky Hunt

**Affiliations:** ^1^ Department of Life Sciences, University of Bath, Bath, BA2 7AY, UK; ^2^ Queen's University Belfast, Belfast, BT9 5DL, UK

**Keywords:** *Strongyloides*, genomics, transcriptomics, proteomics, small RNA, transposable elements

## Abstract

Among nematodes, the free-living model organism *Caenorhabditis elegans* boasts the most advanced portfolio of high-quality omics data. The resources available for parasitic nematodes, including *Strongyloides* spp., however, are lagging behind. While *C. elegans* remains the most tractable nematode and has significantly advanced our understanding of many facets of nematode biology, *C. elegans* is not suitable as a surrogate system for the study of parasitism and it is important that we improve the omics resources available for parasitic nematode species. Here, we review the omics data available for *Strongyloides* spp*.* and compare the available resources to those for *C. elegans* and other parasitic nematodes. The advancements in *C. elegans* omics offer a blueprint for improving omics-led research in *Strongyloides*. We suggest areas of priority for future research that will pave the way for expansions in omics resources and technologies.

This article is part of the Theo Murphy meeting issue ‘*Strongyloides*: omics to worm-free populations’.

## 1. Application of omics, bioinformatics pipelines, functional genomics

The advent of high-throughput, sensitive and cost-effective omics technologies has revolutionized biological research. ‘Omics’ encompasses a range of data types including genomics, transcriptomics, proteomics, peptidomics and metabolomics, which facilitate unbiased, largescale analyses of all of the molecules in a cell, organism or population. In addition, omics also incorporates advanced functional genomics tools, such as RNAi, transgenesis and CRISPR, which can enable a more focused analysis of the function of one, or a few, specific genes of interest. Indeed, for many higher organisms, multi-omics approaches that incorporate multiple omics technologies, tools and data types have become integrated into well-developed platforms that enhance our capability to unravel complex biological questions.

Among nematodes, the free-living model organism *Caenorhabditis elegans* boasts the most advanced portfolio of high-quality omics data that, in tandem with a robust functional omics toolkit, has become one of the most valuable and widely exploited research platforms in biological and biomedical research [1]. While *C. elegans* remains the most tractable nematode and has significantly advanced our understanding of many facets of nematode biology, including parasite systems, the sole use of *C. elegans* as a surrogate system for the study of parasitism and infection biology should be approached cautiously. For example, several parasite-specific gene families are absent or functionally divergent in *C. elegans* [2] hindering the translation of *C. elegans*-derived functional data to parasites. Despite this, the *C. elegans* platform should provide a blueprint for the translation of omics tools and technologies to other nematodes that will pave the way for expansions in omics resources and technologies in therapeutically relevant and experimentally tractable parasitic nematodes [3].

The availability of high-quality species and life cycle stage-specific omics datasets forms the foundation of robust omics platforms. The accessibility and quality of parasitic nematode omics data are continually improving, providing large-scale, rich datasets ripe for exploitation. Indeed, WormBase ParaSite now provides a centralized repository for 177 nematode genomes (WBPS18, April 2023; https://parasite.wormbase.org/index.html) and a range of transcriptome resources, consistently updating and improving assemblies and annotations where possible [4]. In addition to the provision of high-quality omics data, standardized bioinformatics pipelines are also essential to foster a consistent approach to omics analysis that will, in turn, seed downstream functional omics studies in tractable model systems; we are beginning to see the parasitology community recognizing this through the provision of gold standard experimental workflows for helminths [5].

The translation and application of omics approaches to parasitic species continue to be challenged by the nature of the parasitic lifestyles where the inaccessibility of parasite material and difficulty in maintaining life cycle stages *in vitro* hinder progress. Regardless, several species, including *Ascaris suum*, *Brugia malayi* and *Strongyloides* spp., possess traits that have driven their emergence as forerunners in the translation and application of omics technologies to nematode parasites [3,6]. Among these, *Strongyloides* spp. are rapidly becoming the most advanced parasitic nematode model system available, boasting an omics toolkit (including RNAi, transgenesis and CRISPR/Cas9-mediated targeted mutagenesis capabilities) that is currently unrivalled by any other nematode parasite (reviewed extensively elsewhere; see [6]). Further, recent advances in the quality *Strongyloides* omics data also present new opportunities to integrate multiple omics levels (multi-omics) and rapidly progress understanding of parasite biology by connecting genotype and phenotype data in a therapeutically relevant parasitic nematode.

Sustained investment in the development of parasite omics platforms and multi-omics analyses for *Strongyloides* and other nematodes causing Neglected Tropical Diseases will revolutionise the ability to perform comparative omics, evolution, drug resistance and molecular interaction studies that will drive the discovery of new targets for therapeutic intervention, vaccine development and improved diagnostics. Here, we provide an overview of the omics technologies and datasets currently available for *Strongyloides* species, and discuss approaches to data acquisition, data analysis, challenges and future directions.

## 2. *Strongyloides* genome assemblies and annotation

Published genome assemblies are available for four *Strongyloides* species—two parasites of rats, *S. ratti* (44 Mb) and *S. venezulensis* (44 Mb), the livestock parasite *S. papillosus* (61 Mb) and the human parasite *S. stercoralis* (43 Mb)*.* There are also genome assemblies available for two closely related species—a parasite of possums, *Parastrongyloides trichosuri* (43 Mb), and a free-living species, *Rhabditiophanes ditinus* (45 Mb) [7]. While originally the genomes of all six species were sequenced using short-read technologies, four of them: *S. ratti*, *S. stercoralis*, *S. venezulensis* and *R. ditinus*, have since been improved using Oxford Nanopore Technologies (ONT) long-read sequencing [8] (NCBI GCA_029582065.1). *S. ratti*, *S. venezulensis* and *R. ditinus* genome assemblies also incorporated Hi-C maps that map long-range interactions between regions of the genome in 3D space, which can be used to further improve the assemblies as chromosomes tend to have more contacts with parts of the genome that are physically close in the genome [9]. Contiguity of the genome assemblies varies widely; *S. venezulensis* and *R. ditinus* both have chromosome-length scaffolds, and *S. ratti* has two autosomal scaffolds and the X chromosome is in two scaffolds. The assembly of *S. stercoralis* has two autosomes at chromosome-level scaffolds and the X chromosome is in six scaffolds [8] (NCBI GCA_029582065.1). The genome assemblies for *S. papillosus* and *P. trichosuri* are more fragmented due to only being based on short-read Illumina sequencing, and contain 4703 and 1810 scaffolds, respectively, which, in contrast to the other species, lack assignment to chromosomes (table 1). A custom pipeline for *Strongyloides* gene model annotation was built around AUGUSTUS [19] and MAKER [20] training tools using manual annotations of between 197 and 423 genes (depending on species), RNA-seq data and genes from closely related species. This resulted in the annotation of 12 474, 16 904, 18 456 and 13 123 genes in *S. ratti*, *S. venezulensis*, *S. papillosus* and *S. stercoralis*, respectively [7].

**Table 1.** Summary of genome assemblies for *Strongyloides* spp., the closely related species *P. trichosuri* and *R. ditinus*, *C. elegans* and a selection of parasitic nematodes with high-quality genome assemblies.

species	clade	genome size (Mbp)	no. of chromosomes^a^	N50 (Mbp)	no. of scaffolds	no. of annotated protein-coding genes (%)	BUSCO assembly score	data used in assembly	reference
***Strongyloides* species**									
*S. ratti*	IV	43.9	3 (1)	12.1	4		87.0	Illumina, ONT, Hi-C	Kounosu *et al.* [8]
*S. ratti*	IV	43.17	3 (1)	11.7	136	12 464	78.1	genetic map, Illumina, Sanger, 454 pyrosequencing	Hunt *et al.* [7]; Nemetschke *et al*. [10]
*S. stercoralis*	IV	42.7	3 (1)	11.7	8		86.6	ONT	NCBI accession: GCA_029582065.1
*S. stercoralis*	IV	42.7	3 (1)	0.4	800	13 123	78.5	Illumina	Hunt *et al.* [7]
*S. venezulensis*	IV	44.4	2 (0)	31.3	2		85.5	Illumina, ONT, Hi-C	Kounosu *et al.* [8]
*S. venezulensis*	IV	52.2	2 (0)	0.7	587	16 904	76.7	genetic map, Illumina	Hunt *et al.* [7]
* S. papulosis*	IV	60.5	2 (0)	0.1	4703	18 456	77.2	Illumina	Hunt *et al.* [7]
**closely related species and *C. elegans***									
*P. trichosuri*	IV	42.5	3 (1)	0.8	1810	15 010	78.1	Illumina	Hunt *et al.* [7]
*R. ditinus*	IV	44.9	6 (1)	7.8	6		86.6	Illumina, ONT, Hi-C	Kounosu *et al.* [8]
*R. ditinus*	IV	47.3	5 (0)	0.5	471	13 496	74.1	Illumina	Hunt *et al.* [7]
*C. elegans*	V	100.3	6 (1)	17.5	6	19 981	98.6	genetic map, Sanger	the *C. elegans* Sequencing Consortium [11]
**other parasitic nematodes**									
*A. suum*	III	278.6	24 (5)	6.4	108	16 778	93.8	PacBio, Hi-C	Wang *et al.* [12]
* B. malayi*	III	88.2	5 (2)	14.2	205	10 878	96.8	optical map, Illumina, Sanger, 454 pyrosequencing, PacBio	Tracey *et al.* [13]
* B. xylophilus*	IV	78.3	6 (0)	12.8	11	15 884	77.7	Illumina, ONT, Hi-C	Dayi *et al.* [14]
*H. contortus*	V	283.4	6 (1)	47.4	7	19 623	86.6	optical map, genetic map, Illumina, 454 pyrosequencing, PacBio, 10× linked long-range	Doyle *et al.* [15]
*H. glycines*	IV	158	9 (0)	17.9	2121	22 465	55.2	Illumina, PacBio, Hi-C	Masonbrink *et al.* [16]
*O. volvulus*	III	96.4	5 (2)	25.5	1006	12 109	97.6	optical map, Illumina	Cotton *et al.* [17]
*T. muris*	I	111.8	4 (2)	25.5	803	14 995	69.9	Illumina, 454 pyrosequencing	Foth *et al.* [18]

^a^The numbers of chromosomes that are sex chromosomes are indicated in brackets.

### (a) Comparison with *Caenorhabditis elegans*

*Caenorhabditis elegans* was the first multicellular organism to have its genome assembled. This was achieved using a genetic map built during the 1980s and Sanger capillary sequencing with targeting of regions without a known sequence, resulting in an essentially complete assembly, with only a few known gaps, that has been used for most subsequent research [11]. Comparative analysis with a long read-based assembly suggests this is 99.98% identical to current *C. elegans* genomes, having missed only 1.8 Mb of repetitive regions [21]. Compared to *Strongyloides* spp., the *C. elegans* genome is 100.29 Mb, more than double the 43.9 Mb *S. ratti* genome, and is comprised of six chromosomes, compared to the three chromosomes for *S. ratti* and *S. stercoralis* and two chromosomes for *S. papillosus* and *S. venezuelensis*. The *C. elegans* genome also has a higher number of annotated genes—19 981 (WormBase Release 289)—compared to *S. ratti's* 12 474. The N50 size is also larger (17.5 Mb for *C. elegans* versus 12.1 Mb for *S. ratti*); however, this is likely due to the larger size of the *C. elegans* genome, which has longer chromosomes (size range 12–20 Mb versus 12–18 Mb for *S. ratti*) (table 1).

### (b) Comparison with other parasitic nematodes

*S. ratti* has had one of the most contiguous genome assemblies among parasitic nematodes since the assembly became available in 2016 [22]. However, in recent years, new assemblies with near chromosome-length contigs have been created for parasitic nematodes such as *A. suum* [12], *Haemonchus contortus* [15], *Bursaphelenchus xylophilus* [14], *B. malayi* [13] and *Heterodera glycines* [16]*.* Like the most recent 2023 *Strongyloides* genome assemblies, these assemblies have taken advantage of long-read sequencing (e.g. ONT or PacBio HiFi) and Hi-C contact data (table 1). Annotation methods vary between genome assemblies and have mostly been generated using in-house pipelines incorporating programs such as AUGUSTUS [23] and MAKER [20]. Gene model annotations vary between 10 878 and 22 465 annotated protein-coding genes in the genome (table 1).

### (c) Future areas for priority

(i) Improvement of existing reference genome assemblies of *S. papillosus* and *P. trichosuri* using long-read sequencing and Hi-C contact maps to scaffold is a priority. This will generate assemblies with chromosome length contigs that would open opportunities for comparative genomic analysis such as evaluating the role of gene organization in infection. (ii) Genome sequencing of more *Strongyloides* species, particularly those that are medically important such as the human parasite *S. fuelleborni*, to improve genome-level understanding of therapeutically relevant parasitic nematode species. (iii) Current genome assemblies are limited to laboratory-based cultures. *S. ratti* and *S. stercoralis* have been maintained in the laboratory since 1960 (M Viney 2023, personal communication) and 1984 [24], respectively. High-quality sequencing and assembly of wild isolate genomes is important for understanding the genomics of *Strongyloides* in real-world infections. Combining these data with clinical information or information about infectivity is also important to determine the genetic basis of *Strongyloides* parasitism and addressing questions around the zoonotic characteristics of *S. stercoralis*.

## 3. *Strongyloides* phylogenetics, phylogenomics and population genetics and genomics

Although not strictly an omics application, phylogenetics and population genetics are useful tools for exploring evolutionary histories and relatedness across *Strongyloides* samples within a population (population genetics) and between species (phylogenetics). Phylogenomics and population genomics, i.e. where information from genome sequences are used instead of information for a single or a small selection of genes or sequences, is fast becoming a popular method to better study understand relatedness and differentiation within and between populations and species. Here, we summarize the research on *Strongyloides* using both genetic and genomic-based approaches. Most phylogenetics datasets for *Strongyloides* focus on the epidemiology of *S. stercoralis* or *S. fuelleborni* in two key areas: (i) infection surveillance to track transmission in areas where *Strongyloides* is endemic and (ii) zoonotic transmission, to identify shared haplotypes of parasites infecting both humans and other animals (particularly non-human primates and dogs; table 2). Such studies are largely based on reconstructing phylogenies or categorizing haplotypes using mitochondrial DNA sequences, including the 18S rRNA subunit or the *cox1* (cytochrome c oxidase subunit 1) gene [49]. The hypervariable regions of the 18S small subunit [50] have species-specific arrangements and facilitate differentiation between *Strongyloides* species.

**Table 2.** Summary of phylogenetics, population genetics, phylogenomics and population genomics studies on *Strongyloides* species. SSU, small subunit; LSU, large subunit.

species	host	genetic marker(s)	reference
**marker gene studies**			
*S. ratti*	*Rattus norvegicus*	Actin, BSP-8, CM-2	Fisher & Viney [25]
*S. ratti*	*Rattus norvegicus*	Actin, BSP-8	Paterson *et al*. [26]
**mtDNA studies**			
*S. papillosus*, unknown *Strongyloides* of cows (suggested name *S. vituli*)	*Ovis aries*	18S SSU	Eberhardt *et al.* [27]
	*Bos taurus*		
unknown *Strongyloides* infections of humans, *S. stercoralis, S. fuelleborni, S. planiceps*	*Homo sapiens*	18S SSU, *cox1*	Hasegawa *et al.* [28]
	*Canis familiaris*		
	primate (various)		
*S. fuelleborni*	*Papio* sp*.*	18S SSU	Anderson *et al.* [29]
*S. stercoralis*	*Homo sapiens*	18S SSU	Schär *et al.* [30]
*S. stercoralis*	*Canis familiaris*	18S SSU, 28S SSU, *cox1*, *MSP* gene	Nagayasu *et al.* [31]
	*Homo sapiens*		
*S. stercoralis, S. fuelleborni*	*Papio papio*	18S SSU	Barratt *et al.* [32]
	*Canis familiaris*		
	*Homo sapiens*		
*S. stercoralis*	*Canis familiaris*	18S SSU, *cox1*	Basso *et al.* [33]
*S. stercoralis*	*Canis familiaris*	18S SSU, *cox1*	Beknazarova *et al.* [34]
	*Homo sapiens*		
*S. stercoralis*	*Canis familiaris*	18S SSU, *cox1*	Sanpool *et al.* [35]
	*Homo sapiens*		
*S. stercoralis*	n.a.	*cox1*, retrieved from published data repositories	Spotin *et al.* [36]
*S. fuelleborni*	*Macaca fascicularis*	18S SSU, *cox1*	Thanchomnang *et al.* [37]
*S. stercoralis, S. tunefaciens*	*Felis catus*	*cox1*	Wulcan *et al.* [38]
*S. stercoralis*	n.a.	18S SSU and *cox1* retrieved from published data repositories	Barret & Sapp [39]
*S. fuelleborni*			
*S. fuelleborni*	*Macaca nemestrina,*	Am	Janwan *et al.* [40]
	*Homo sapiens*		
*S. stercoralis, S. procyonis*, *S. planiceps*, unknown *Strongyloides*	*Procyon lotor*	18S SSU, 28S LSU, *cox1*	Ko *et al.* [41]
	*Meles anakuma*		
	*Nyctereutes procyonoides Paguma larvata Mustela sibirica*		
	*Felis catus*		
*S. stercoralis*	*Homo sapiens*	*cox1*	Repetto *et al.* [42]
*S. fuelleborni*	*Macaca mulatta*	18S SSU, *cox1*	Ko *et al.* [43]
also found*: S. cebus, S. vituli*			
*S. fuelleborni*	*Macaca mulatta*	18S SSU, *cox1*, complete mitochondrial genome	Ko *et al.* [43]
*S. vituli*			
*S. cebus*	*Macaca fuscata*		
	*Trachypithecus francoisi*		
	*Pygathrix nemaeus*		
	*Pongo pygmaeus*		
	*Symphalangus syndactylus*		
	*Saimiri boliviensis*		
**whole genome shotgun studies**			
*S. stercoralis*	*Homo sapiens*	whole genome shotgun	Kikuchi *et al.* [44]
*S. stercoralis*	*Canis familiaris*	18S SSU, *cox1*, whole genome shotgun (subset)	Jaleta *et al*. [45]
	*Homo sapiens*		
*S. stercoralis*	*Homo sapiens*	18S SSU, *cox1*	Zhou *et al.* [46]
		whole genome shotgun (subset)	
*S. stercoralis*	*Homo sapiens*	18S SSU, *cox1*, whole genome shotgun (subset)	Aupalee *et al.* [47]
*S. ratti*	*Rattus norvegicus*	whole genome shotgun	Cole *et al.* [48]

More recently, there has been an increase in the use of phylogenomics and population genomics, using shotgun genome sequencing and evaluation of SNPs to try to resolve more detailed phylogenies and thereby improve understanding of how *Strongyloides* species evolve, and identify differences in genomic variation within and between populations and species. Population genomics has not been explored deeply in *Strongyloides* spp. Two studies have performed whole genome shotgun sequencing and examined genetic diversity in *S. stercoralis* isolates from infected patients [44,46]. Both studies found geographical clustering of the samples based on their genomes and little evidence of recombination, suggesting that the sexually reproducing free-living stage is rare in the wild populations. A population genomics study on *S. ratti* from wild rat faeces collected in England and Wales found that a few long-lived lines infect many rats across the three distinct sampling sites studies and there was little variation between the sites [48]. There is a strong positive selection pressure, with a high number of non-synonymous SNPs, on gene families previously associated with *Strongyloides* parasitism in *S. ratti* (astacin-like metalloendopeptidases, CAP-domain containing proteins and acetylcholinesterases) [48]. For all of these studies, only samples from a single geographical area (East Asia and the UK, respectively) are used so they are unlikely to be capturing all of the genetic diversity of either *Strongyloides* species.

### (a) Comparison with *Caenorhabditis elegans*

Recently, *C. elegans* population genomics has been used to (i) discover novel genotypes and unravel the genetic underpinnings of phenotypes [51]; (ii) investigate the evolution of hermaphrodism and the mating system [52]; (iii) assess genetic effects on the starvation response [53]; and (iv) assess genetic diversity and adaption to different environments [54]. There have also been studies on *C. elegans* laboratory strains to, for example, investigate experimental evolution [55] and study evolutionary responses to bacteria [56]. Most *Strongyloides* research in this area is solely concerned with transmission or species specificity; therefore, these *C. elegans* applications are much more diverse in comparison. There is scope to expand the types of questions and analyses carried out in *Strongyloides* studies, for example by focusing on other features of their life history or ecology and evolution.

### (b) Comparison with other parasitic nematodes

Phylogenetics studies have long been carried out on parasitic nematodes using marker genes, and more recently this has extended to phylogenomics and population genomics studies from whole genome sequencing data [57]. These studies span nematodes that infect plants [57], wild animals [58] and humans [59]; they are more diverse than those conducted in *Strongyloides*, addressing a wide range of topics including identifying a genetic locus associated with anthelminthic drug resistance in multiple distinct populations of *H. contortus* [60], and understanding zoonotic reservoirs and distribution expansion of *Trichuris trichiura* [61].

### (c) Future areas for priority

A priority is to perform population genomics studies for a wider range of *Strongyloides* species and over a wider geographical distribution of sampling sites. This would provide information about which regions of the genome are under more evolutionary pressure and how the populations in endemic areas are linked or associations of genetic signatures with anthelminthic drug resistance. This knowledge could be useful when selecting vaccine or drug targets [62], as well as providing insights into the evolution of parasitism. Additionally, this would allow identification and monitoring of antihelminth resistance variants in populations, which is an important goal in the World Health Organisation's Roadmap [63]. Improving our understanding of the distribution of *Strongyloides* infection genotypes, and matching this with information on infection phenotypes, e.g. severity of infection or symptoms associated with infection, should be a key focus for this research area. This information could facilitate the prediction of important information associated with infection including transmission dynamics, disease severity and treatment susceptibility. These insights would provide both epidemiological and clinical benefits, resulting in improved modelling of transmission and development of genomic tests to identify more appropriate treatments. We also need to diversify the range of *Strongyloides* species studied as the majority of published studies focus on human-infecting species. Generating data from a broader range of *Strongyloides* species, such as *S. papillosus*, would aid the creation of similar models for livestock treatment. In species such as *S. ratti*, whole genome population data would additionally facilitate complementary laboratory-based experiments e.g. to test hypothesizes about genotype–phenotype associations.

## 4. *Strongyloides* transcriptomics

**Figure 1.**
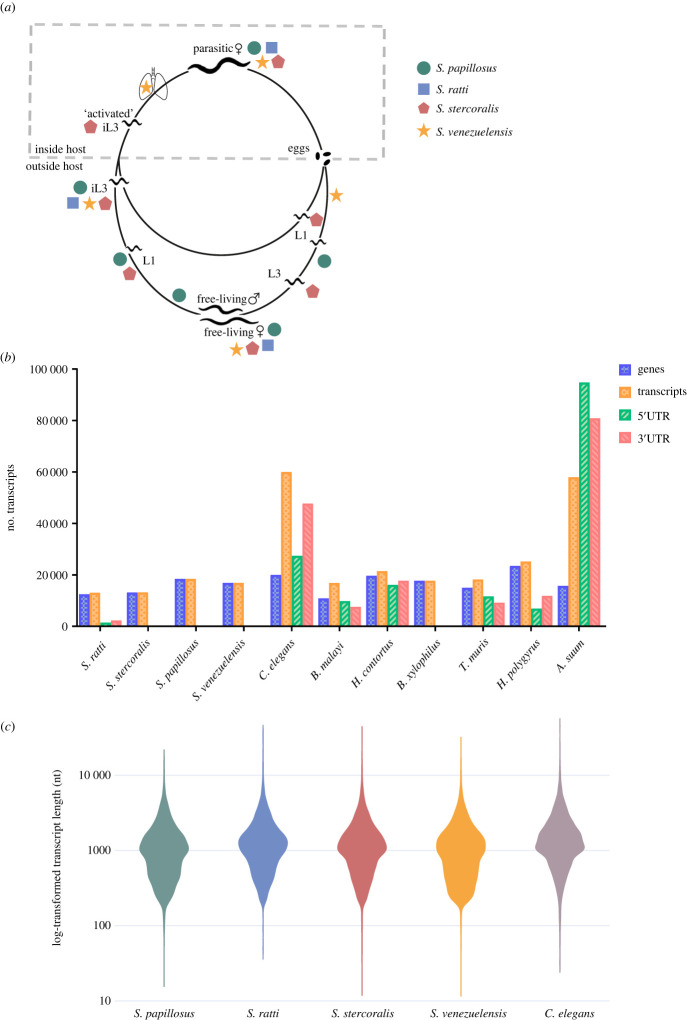
Transcriptome information for the *Strongyloides* species. (*a*) Availability of transcriptome data divided by life cycle stage for *S. papillosus* (green circle), *S. ratti* (blue square), *S. stercoralis* (red hexagon) and *S. venezuelensis* (yellow star) [7,66–69]. (*b*). Number of transcripts, 5′UTR and 3′UTR annotations for 11 nematode species including four *Strongyloides* spp. Note that multiple UTR annotations are sometimes reported for a single transcript. (*c*) *S. papillosus*, *S. ratti*, *S. stercoralis*, *S. venezuelensis* and *C. elegans* comparison of log-transformed transcript length. Transcript length is significantly different between all pairwise comparisons apart from *S. ratti* and *S. stercorali*s, and *S. venezuelensis* and *S. papillosus* (ANOVA, *F* = 485.7, *p* < 0.0001; Games–Howell multiple comparison test: Sr–Ss md = 65.72, *p* = 0.0013; Sr–Sp md = 221.9, *p* < 0.0001; Sr–Sv md = 234.4, *p* < 0.0001; Sr–Ce md = −238.2, *p* < 0.0001; Ss–Sp md = 156.2, *p* < 0.0001; Ss–Sv md = 168.6, *p* < 0.0001; Ss–Ce md = −303.9, *p* < 0.0001; Sp–Sv md = 12.42, *p* = 0.8606; Sp–Ce md = −460.1, *p* < 0.0001; Sv–Ce md = −472.5, *p* < 0.0001). (Online version in colour.)

The transcriptome represents a portion of the genome transcribed into RNA molecules (expressed) in a given tissue, life cycle or organism [64] and by comparing transcriptomes we are able to identify the genes that are up- or downregulated in different life cycle stages or conditions. Transcripts are composed of introns, exons, 3′ and 5′ untranslated regions (UTRs), a 5′ cap and a 3′ poly A tail. Using the 454/Roche pyrosequencing technology [65] the transcriptomes of four developmental stages of *S. venezuelensis* were sequenced [66]. The majority of differentially expressed genes were in parasitic adults (226 genes) compared to eggs/L1, third stage infective larvae (iL3s) and lung-stage larvae (33, 68 and 37 genes, respectively; figure 1*a*). Genes coding for nicotinamide phosphoribosyltransferase, tuberin and ferrochelatase were identified as putative parasitism-related genes. *Strongyloides* spp. exhibit a unique life cycle in which genetically identical parasitic adult female (pF) and free-living adult female (flF) generations exist. These life cycle stages can be directly compared to investigate the genes underpinning parasitism; for example, genes significantly upregulated in the pFs versus flFs can identify genes putatively involved in parasitism or other features associated with the parasitic life stage [7]. RNAseq data were analysed using this comparative approach for *S. stercoralis*, *S. ratti*, *S. papillosus* and *S. venezuelensis* [7,67–69] (figure 1*a*). In total, 10–18% of expressed genes were identified as differentially expressed between flFs and pFs in the four *Strongyloides* species. Across *Strongyloides* spp., genes from 29 families are upregulated in pFs and are thought to have a putative role in parasitism; including genes that encode acetylcholinesterase, astacin-like metalloendopeptidase and CAP-domain containing proteins (also referred to as SCP/TAPs) [68,69].

Of the 1188 genes in *S. stercoralis* and 909 in *S. ratti* which were specifically upregulated in pFs compared with flFs, 18–19% code for astacin-like metalloproteases or CAP-domain containing proteins [69]. The types of gene families upregulated in the pF (*cf.* flF) of *S. stercoralis*, *S. ratti*, *S. papillosus* and *S. venezuelensis* differs between species. Specifically, 25–305 gene families were uniquely upregulated in pFs of one species but not the pFs of the other three *Strongyloides* species. *S. venezuelensis* is the most divergent and 305 gene families containing 327 genes upregulated in the pF (compared to the flF) are unique to this species [68], suggesting different species may use distinct molecular toolboxes to infect their host. Interestingly, the upregulated parasitism-associated gene families are expanded in *Strongyloides* spp. coinciding with the evolution of parasitism within the *Strongyloides* clade. Also, genes upregulated in the *S. ratti* pF, compared with the flF or iL3 stage, are physically clustered together on regions of chromosome II, which could be important for the co-regulation of transcription of parasitism-associated genes [69].

The transcriptome of the iL3 life cycle stage has provided further insight into the genetic basis of *Strongyloides* parasitism. The iL3 has both a free-living stage where it is seeking a host, and a parasitic stage where it penetrates and migrates through host tissue en route to the small intestine. iL3s have been recovered from host lungs and the lung is believed to be a common route of infection for iL3s migrating to the small intestine. After the intestine, the lungs are the most common organ to find *Strongyloides* iL3s located during a disseminated infection [70]. The iL3 lung stage in *S. venezuelensis* has been associated with an upregulation of six astacin-like metalloproteases and two glycoprotein coding genes, predicted to code for proteins in excretory/secretory products [66]. In general, much less is known about the genes involved during migration or disseminated infection in the lungs compared with other life cycle stages. The transcriptome varies between the parasitic and free-living larval stages in both *S. venezuelensis* and *S. stercoralis* and based on their transcriptome, larval stages appear to be separated by life cycle stage rather than species, despite *S. venezuelensis* and *S. stercoralis* infecting different hosts (rats and humans, respectively) [71].

RNAseq transcriptomics of seven life cycle stages of *S. stercoralis* [67] was used for comparative analysis of developmental larval forms of *Strongyloides* and *C. elegans*, to investigate similarities of molecular regulation of developmental arrest between the *C. elegans*' dauer larvae and parasitic iL3s [71,72]. Genes encoding components in the cGMP pathway (which controls dauer arrest in *C. elegans*) were upregulated in *Strongyloides* iL3s, compared to other larval stages. In addition, gene homologues of *C. elegans* dauer arrest and activation insulin-like signalling, and TGFβ signalling, are also upregulated in *S. stercoralis* iL3s, suggesting a role in iL3 development. In *S. papillosus*, transcript expression analysis of 10 RNAseq datasets across six life cycle stages indicates a high degree of conservation in developmental regulation across *Strongyloides* species [69]. The majority of *S. papillosus* genes are developmentally regulated with 73% of genes differentially expressed across life cycle stages; 10% of *S. papillosus* genes were differentially expressed between pFs and flFs, 21% were differentially expressed between free-living males (flM) and flFs and 42–45% were differentially expressed between flF/pF and iL3s. In a comparative transcriptomic analysis, 55% of orthologues from *S. venezuelensis* and *S. stercoralis* were assigned to different developmental stages including eggs, larvae and adult stages [71].

Investigation of genes that are differentially expressed when *Strongyloides* spp. are exposed to different host conditions contributes to our understanding of *Strongyloides* survival and adaptation to their host [66,73–75]. In pF *S. ratti*, genes involved in collagen regulation, muscle function and repair, are upregulated in immunized versus naive mice, indicating that the *S. ratti* transcriptome can be altered to facilitate protection against damage and expulsion from the host [73]. However, the mouse is not a natural host of *S. ratti* and this must be taking into consideration when interpreting these results. A comparison of gene expression in *S. ratti* pF derived from the natural rat host compared with pFs derived from a permissive gerbil host, demonstrated an increase in astacin-like metalloproteases and acetylcholinesterase genes in the gerbil-derived parasites further highlighting a putative link between astacin-like metalloprotease expression and parasitism. Even though fewer iL3s successfully establish infection in gerbils, gerbil-derived *S. ratti* parasitic adults produce more male larvae and survive/reproduce for longer, thus highlighting differences in *S. ratti* survival and reproduction when exposed to different hosts [74].

There is a lack of information about how *Strongyloides* spp. respond at the transcriptome level to drug treatments. When comparing human patients' faecal samples cultured on nutrient agar plates that are either untreated or treated with the corticosteroid dexamethasone (DXM), free-living adult stages of *S. stercoralis* display differential expression of 199 and 263 genes upregulated in flFs and flMs, respectively, that are involved in developmental processes, multicellular organismal processes and embryonic morphogenesis [75]. This raises questions about how clinical steroids and other medication can affect parasite development and survival in endemic strongyloidiasis. As we continue to generate and collate *Strongyloides* transcriptome data, data repositories and software such as the *Strongyloides* RNA-seq Browser [76] will be useful in combining existing data from *Strongyloides* species and will facilitate easier comparative gene expression and enrichment analyses.

### (a) Comparison with *Caenorhabditis elegans*

The *C. elegans* transcriptome (PRJNA13758) contains 31 989 transcript variants for 19 981 protein-coding genes with further 28 138 transcripts for other RNA sequences such as pseudogenes and non-coding RNAs. In *Strongyloides* spp*.* the total number of transcripts annotated is similar to the total number of genes (figure 1*b*) (WormBase ParaSite Version WBPS18) [4] because the transcript variants for *Strongyloides* genes are not well annotated. Furthermore, while most transcripts have an average read length of 1202–1237 bp across all four *Strongyloides* species sequenced, similar to *C. elegans*, there are an increased number of shorter length transcripts in *Strongyloides* spp. especially in *S. papillosus* and *S. venezuelensis* (figure 1*c*). This may represent genuinely shorter transcripts in *Strongyloides* spp. but is likely to be indicative of annotations that require improvement. Information on the UTR regions of transcripts is also lacking for *Strongyloides* spp. *C elegans* remains at the forefront of nematode transcriptome data availability and annotation quality and is continuously improving through sustained contributions from a large scientific community. Current *C. elegans* annotations contain 27 371 5′UTR and 47 676 3′UTRs (n.b. some transcript variants have multiple UTR annotations). In *C. elegans*, long-read cDNA and direct RNA sequencing has further improved transcriptome annotations. Using ONT long-read sequencing 23 865 isoforms have been confirmed from 14 611 genes, out of which 3452 were previously undiscovered, with 16 342 isoforms in the 3′UTR region and polyA identification and annotation in all the major life cycle stages, excluding embryos and the arrested dauer stage [77]. ONT sequencing has also been applied to *C. elegans* to further improve the transcript annotations, identify trans-splicing of mRNAs and confirm splice leaders 1 and 2 (SL1, SL2) [78].

### (b) Comparison with other parasitic nematodes

With the improved availability and affordability of RNAseq, transcriptome data is available for many parasitic nematodes, especially those from the nematode clades III–V [79–82]. The availability and quality of stage-specific expression varies between parasitic nematode species, with adult life cycle stages more likely to be sequenced and egg stages the least likely [79]. While many parasitic UTRomes and alternative splicing annotations remain unexplored, with the advance of RNAseq there has been a recent increase in UTR and splicing annotations. *Brugia malayi* (PRJNA10729), *Heligmosomoides polygyrus* (PRJEB15396) and *Trichuris muris* (PRJEB126) have transcript variant data available, and 27–64% of transcripts contain UTR annotations (WormBase ParaSite Version WBPS18). In *A. suum*, over 60 000 transcripts have been annotated and validated from RNAseq data, defining alternatively spliced and trans-spliced transcripts and allowing for an extended annotation of UTR regions of previously annotated transcripts [83]. Using PacBio Iso-seq, transcriptome annotations and isoform classification have been improved for *H. contrortus* [15], however, most parasitic nematode species do not have long-read transcriptome data available. In *H. contrortus* 67.8% of transcripts were annotated with UTR regions following long-read sequencing and manual curation, many of which were previously misannotated as coding exons [15,22], further highlighting the importance of long-read sequencing and high-quality annotations.

### (c) Priorities for future work

There is limited egg and early stage host-migrating larvae transcript information available for *Strongyloides* spp. and other parasitic nematodes. In addition, time point comparisons within life cycle stages have demonstrated differential expression [73] but these data are lacking for most life cycle stages and species. Improved annotation of transcripts including identifying transcript variants is essential to improve gene expression and regulation studies, and for functional genomic applications. Because conventional RNAseq methods use short RNA fragments (approx. 50–150 nt) and are mainly optimized for sequencing protein-coding regions of genes, there is a lack of precision in defining ends of genes and information on alternative splicing and UTR regions [84]. While UTR sequencing (3P-Seq) and pipelines for UTR prediction from RNAseq data are available, these methods are technically challenging, require separate library preparations, mainly focus on 3′UTR regions and can be difficult outside of model organisms [85]. Additionally, short-read sequencing can cause mapping errors of exons and UTRs which fails to recognize alternatively spliced isoforms of transcripts and UTRs. Data on 5′UTR and 3′UTRs, which play an importance in gene regulation, is missing entirely from *S. papillosus*, *S. venezuelensis* and *S. stercoralis* (figure 1*b*) and partially from *S. ratti* for which we have 5′UTR and 3′UTR annotation for only 11% and 17% of the transcriptome (figure 1*b*). Improved UTR data will enable us to bioinformatically more accurately predict target sites for small RNAs (sRNAs) and to improve transcript comparisons between life cycle stages, e.g. are different transcript variants of the same gene used in different life cycle stages? Long-read sequencing technologies such as ONT and PacBio are important tools for improving these annotations in the future. Long-read sequencing enables us to sequence full-length transcripts, accurately characterizing the transcripts from 5′UTR to 3′UTR region, including alternatively splicing of exons. Long-read sequences also reduce the GC and amplification bias of RNAseq, improving the quantification of transcripts [86]. Sequencing data from single cell and spatial transcriptomics to map localization of gene expression are also not available either for *Strongyloides* species or for most other parasitic nematodes, but would offer further insights into genes that are important for parasitism and development. The increased availability of sequencing data for more *Strongyloides* species and strains, along with further research into their life cycle stages—including time points within these stages and data on gene expression for wild isolates—will aid our understanding of *Strongyloides* species’ adaptation to their environment and their gene regulation response to environmental change.

## 5. *Strongyloides* proteomics

Proteomics is an important tool for understanding the molecular basis of parasitism, where the application of proteomics tools has the potential to characterize protein fingerprints associated with parasite infection, development and parasite–host–microbe interactions. With the advent of tandem liquid-chromatography–mass spectrometry (LC–MS/MS) and improvements in nematode genomic and transcriptomic resources, research into nematode proteomics has expanded in recent years. Most of the current nematode proteomics research can be divided into three main categories based on the source material: (i) somatic (whole-worm) lysate, (ii) excretory–secretory products (ESPs) and (iii) extracellular vesicles (EVs).

Nematode somatic proteomes are typically derived from whole-worm lysates whereby nematode tissue has been mechanically disrupted, resuspended in a lysis buffer and subjected to protein extraction. Most of the somatic proteomics research in *Strongyloides* spp. has focused on the iL3 stage to better understand strategies used to establish infection [7,87–89] (table 3). The first major proteomics study in *Strongyloides* spp*.* was conducted with *S. stercoralis*, followed by studies in *S. ratti* [87] and *S. venezuelensis* [88]. The seminal *S. stercoralis* proteomics study used iL3s obtained from infected patients and identified 26 proteins including myosin, actin, elongation factors, ATP synthases, galectin and stress response proteins [90]. However, one limitation of this work was that a large portion of mass-spectrometry (MS) spectra remained unassigned following LC–MS/MS data analysis; this may be explained by the unavailability of genomic information and expressed-sequence tag (EST) datasets at the time of the study [90]. The release of the *S. stercoralis* draft genome in 2016 [7] enabled Dishnica and colleagues [89] to build upon the *S. stercoralis* iL3 proteome to identify 430 proteins (3.3% of the predicted protein dataset) in *S. stercoralis* iL3 whole-worm lysates [89] including 3 of the 4 major antioxidant families previously identified in the LC/MS analysis of *S. ratti* [87] and *S. venezuelensis* [88] ESP (table 3). Many of the protein categories proposed to be involved in iL3 parasitism [7] were also identified, for example astacin-like metalloproteases that have been reported to be fundamental to the initial phases of *S. stercoralis* host tissue penetration, parasite development and host immune evasion [96]. Overall, this LC–MS/MS analysis supports the predictions from other *Strongyloides* spp. transcriptomic, genomic and proteomic analyses by confirming the presence of proteins that were predicted to be associated with *Strongyloides* spp. parasitism. Collectively the data derived from the above *Strongyloides* spp. studies highlight the benefits of a multi-omics approach whereby the combination of *in silico* approaches (genomic and transcriptomic) with experimental proteomics (LC–MS/MS) has the potential to identify proteins associated with parasitism and reveal complex host–parasite interactions, reviewed here and also elsewhere in this issue [97].

**Table 3.** Summary of *Strongyloides* proteomics studies. ESP, excretory/secretory products; iL3, infective L3 larvae; fLF, free living female; pF, parasitic female.

species	life cycle stage	sample type	reference
*S. stercoralis*	iL3	somatic extract—whole worm lysate	Marcilla *et al.* [90]
*S. stercoralis*	iL3	somatic extract—whole worm lysate	Rodpai *et al*. [91]
*S. stercoralis*	iL3	somatic extract—whole worm lysate	Dishnica *et al.* [89]
*S. stercoralis*	L3	somatic extract—whole worm lysate	Rodpai *et al*. [92]
*S. ratti*	iL3, flF, pF	ESP	Soblik *et al.* [87]
*S. ratti*	pF	ESP	Younis *et al.* [93]
*S. ratti*	flF, pF	somatic extract—whole worm lysate	Hunt *et al.* [7]
*S. venezuelensis*	iL3s, pF	ESP	Maeda *et al.* [88]
*S. venezuelensis*	iL3	somatic extract—whole worm lysate	Fonseca *et al.* [94]
*S. venezuelensis*	iL3	somatic extract—whole worm lysate	Corral *et al.* [95]

There is a demand for the identification of new biomarkers for novel *Strongyloides* diagnostics [98]. Proteomics has the potential to identify proteins with potential immunogenic properties that may serve as biomarkers for novel diagnostic tests. Previous somatic proteomic analysis of *S. venezuelensis* iL3s identified 877 proteins that included numerous proteins with biomarker potential such as antioxidants, astacin-like metallopeptidases, proteases and galectins [94] (table 3). Study [94] also highlighted the need for characterization of CAP domain-containing proteins, which are thought to be immunogenic [99]; indeed, nine proteins, including CAP domain-containing proteins, were also detected in *S. stercoralis* somatic extracts [89]. Interestingly, CAP domain-containing proteins are thought to be associated with parasitism due to their expansion in *Strongyloides* and *Parastrongyloides* spp. but their absence from the closely related free-living species *Rhabditophanes* sp*.*. Comparison of CAP domain-containing protein detection in other parasitic nematode species in combination with functional characterization may shed light on their potential as strongyloidiasis-specific diagnostic markers [7]. Both studies provide a catalogue of potential diagnostic biomarkers for human and animal strongyloidiasis; however, further functional work is required to characterize their immunogenic properties and promise as novel biomarkers.

An emerging area of research is the use of reverse *in silico* approaches to predict putative immunogenic proteins for diagnostic/vaccine development [100]. A similar approach was recently applied to predict 34 immunogenic proteins from *S. stercoralis* proteomics data that hold potential as novel vaccines or diagnostic biomarker candidates [101]. None of the 34 proteins identified in *S. stercoralis* were identified in a previous *S. venezuelensis* LC–MS/MS analysis [94]; this may reflect species differences and/or differences in experimental approaches.

Excretory–secretory products (ESPs), released by helminths into the host environment, are considered an important facet of host–parasite–microbe interactions [102]. As such, ESP characterization will drive an improved understanding of the complexity of the host–parasite–microbe interface [103]. To date *Strongyloides* spp. ESP proteomics analysis has been limited to *S. ratti* and *S. venezuelensis* species. A large scale comparative analysis, performed on *S. ratti* iL3, pF and fLF ESP, revealed a total of 586 proteins across all three life stages with 450, 335 and 219 proteins identified in iL3s, pFs and fLFs, respectively [87] (table 3). Of these 586 proteins, 140 were identified across all three life stages and included proteins such as antioxidants, heat shock proteins, carbohydrate-binding proteins, enolases, galectin, transthyretins and CAP domain-containing proteins [87]. Similarly, in *S. venezuelensis* the highest number of proteins were identified in iL3 ESP (436 in iL3 versus 196 in pF), with CAP domain-containing proteins, galectins and enolases among the most abundant proteins detected [88] (table 3). Notably, the astacin-like metalloproteases and CAP-domain containing proteins were identified in both *S. ratti* and *S. venezuelensis* iL3 ESP and are upregulated (at both the gene and protein levels) in *S. ratti* pFs [7,87,88,104]. Interestingly, CAP domain-containing proteins were also identified in *Ancylostoma caninum* iL3 ESP, where they are associated with the free-living to parasitic lifestyle transition, host invasion and host immune system cross-talk [105]; comparison of other *Strongyloides* spp. iL3 ESP proteomes will shed light on the importance of these proteins in iL3 biology. Further life stage-specific ESP comparisons identified both conserved and species-specific proteins in *S. venezuelensis* and *S. ratti* [7,87,88]. In *S. ratti*, 196, 79 and 35 proteins were unique to iL3s, pFs and fLFs, respectively [87] while in *S. venezuelensis*, 350 proteins were specific to iL3s and 94 proteins specific to pFs [88]. The detection of unique ESP proteins in different life cycle stages highlights the need for this analysis to be translated to other *Strongyloides* and *Parastronyloides* spp. to aid the characterization of proteins that may be key to parasitism or parasite development.

EVs are membrane-bound vesicles (40–1000 nm) [106] that are released into the extracellular space within an organism and also, in the case of parasitic nematodes, into the host environment [107]. EV cargo includes microRNAs, proteins/peptides, lipids and signalling molecules [108] that, if released into the host, may modulate host innate and adaptive host immune responses and/or interact with the host environment [107,108]. EV proteomics tools have not yet been exploited in *Strongyloides* spp.; however, translation of established parasitic nematode EV pipelines [109–111] provides an opportunity to characterize *Strongyloides* spp. EV cargo to reveal novel insights into parasitism, host–parasite communication and identify new diagnostic biomarkers.

### (a) Comparison with *Caenorhabditis elegans*

By exploiting LC–MS/MS and shotgun proteomics, Merrihew *et al.* [112] identified approximately 7000 proteins in *C. elegans* tissue [112]. Following this, Schrimpf *et al.* [113] detected approximately 11 000 proteins representing over half of the predicted proteins present in *C. elegans* [113]. Since then, proteomics studies have contributed to the understanding of *C. elegans* biology, including reproduction and development [114], ageing and longevity [115,116] and innate immunity mechanisms against pathogens [117–119]. One hundred and eighty-four proteins have been identified in *C. elegans* ESP, including transthyretin-like proteins, C-type lectins, proteases and fatty acid binding proteins [120]. Interestingly, while some ESP proteins are common to parasitic and free-living species, others appear to be parasite-specific [120]. A number of proteins (enolase, aldolase, peroxiredoxin, hydroperoxide reductase, thiol-specific antioxidants) are absent in *C. elegans* ESP but present in parasitic nematode ESP, suggesting a common functional role in parasitism [120,121]. Of note, *Strongyloides* spp. pFs appear to secrete unique trypsin-inhibitor like (TIL) domain-containing proteins [87,88,120,122] which have been linked to *Stongyloides* spp. parasitism [7]. Like parasitic nematodes, proteomics data on *C. elegans* EV cargo is lagging behind the somatic and ESP proteome analysis; however, 224 proteins have been identified in *C. elegans* EVs and demonstrated that *C. elegans* EV protein composition differs considerably from whole-worm lysate [123].

### (b) Comparison with other parasitic nematodes

Over the past 10–15 years significant progress has been made in proteomics platforms for identification of nematode somatic proteins that hold potential as biomarkers or therapeutic targets [124]. Life stage-specific somatic proteomics data exist for several parasitic nematode species including *Trichinella spiralis*, *H. contortus*, *H. polygyrus* and *Nippostrongylus brasiliensis* that enable species and life stage comparative analyses [125–130]. Indeed, similar to *Strongyloides* spp., there appear to be a core set of proteins (*ca*. 20% in *T. spiralis* and *ca*. 30% in *H. contortus*) that are conserved between parasite life stages, indicating that the majority of proteins detected appear to be life stage-specific [125,131]. Parasitic nematode ESP proteomics has received a lot of attention. Beyond *Strongyloides* spp., there are ESP proteomic profiles for 20 parasitic nematode species including *A. caninum*, *Necator americanus*, *H. contortus* and *H. polygyrus* [132–136]. Comparative analyses of ESP proteomes across parasitic species from different clades and lifestyles reveal conservation of protein families. For example, CAP domain-containing proteins are some of the most abundant proteins detected across multiple parasitic nematode ESPs [134,135,137–139] and they have been implicated in host invasion and parasite development [137]. Other highly conserved ESP proteins include metallopeptidases, transthyretin-like family proteins, astacin-like metalloproteases and enolases [120]. While progress has been slow for *Strongyloides* spp., numerous studies have characterized EV protein cargo in other parasitic nematode spp. [109,110,140–142]. Similar to the somatic and ESP proteomics data, EV proteomics studies have identified proteins conserved across parasitic nematode species [109,140,142]; however, the reproducibility of EV proteomics datasets can be problematic. For example, two independent analyses of *T. muris* EV proteomes yielded different data [142,143] and this highlights the need for a standardized pipeline for the characterization of nematode derived EVs, as outlined elsewhere [5]. Continued proteome comparisons across relevant parasitic nematodes and life stages will aid the identification of proteins of interest that will seed functional characterization and reveal novel biomarkers and vaccine targets [120,124].

### (c) Priorities for future work

Expansion and improvement of genome and predicted protein datasets will facilitate the growth and enhancement of nematode proteomic data derived from somatic (whole-worm) lysate, ESP and EV sources [7]. The continued advancement in proteomics tools, technologies and analysis pipelines in addition to sustained efforts to translate these to parasitic nematodes will also drive a better understanding of *Strongyloides* spp. biology. Indeed, more generally, the standardization of proteomics methods is also critical to the delivery of experimentally robust datasets that will inform nematode biology. Primarily, the priority for *Strongyloides* spp. should be the generation of equivalent proteomics data for somatic, ESP and EVs across *Strongyloides* spp. and life stages to facilitate robust comparative analyses and drive the discovery of new biomarkers and control targets for strongyloidiasis. These data would also enable interrogation of the *Strongyloides* host–parasite interface and the influence of abiotic/biotic environmental factors on the *Strongyloides* ESP and EV proteomes.

## 6. *Strongyloides* peptidomics

Peptidomics is classed as a subset of proteomics and is defined as the peptides (approx. less than 10 kDa) produced in a cell, tissue or organism at a specific timepoint [144]. Relative to proteomics, peptidomics is in its infancy; peptidomics studies in nematode parasites first emerged in the literature around the early 2000s [144–146] and have not yet advanced to include *Strongyloides* spp.

### (a) Comparison with *Caenorhabditis elegans*

The Schoofs laboratory has developed a high-throughput LC–MS/MS peptidomics approach adapted from *Drosophila melanogaster* for application in *C. elegans*. This has resulted in the identification of a raft of *C. elegans* neuropeptides [147–149] that have also been detected in parasitic nematodes [150,151]. Advances in *C. elegans* peptidomics highlights the need for expanded LC–MS/MS studies in parasitic nematodes that exploit *in silico* analyses [152,153] and interrogate somatic, ESP and EV extracts. In addition, as *C. elegans* peptidomics data parallel advances in LC–MS/MS technology, it will become possible to map specific cell/neuron peptide expression and dynamics to provide novel insights into nematode peptide biology that will also aid our understanding of parasite systems.

### (b) Comparison with other parasitic nematodes

Much of the initial parasitic nematode peptidomics research was focused on the pig parasite *A. suum* [154–157]. Since the advent of high-throughput peptidomics only *H. contortus* has been studied in addition to *A. suum*, and the focus has primarily been the identification of neuropeptide and antimicrobial peptide profiles in somatic extracts [150,151,158]. Integrating *in silico* genomics and transcriptomics with LC–MS/MS has further advanced proteomic and peptidomics research [159], and these approaches are beginning to emerge within parasitic nematode literature [160].

### (c) Priorities for future work

The advancement of *Strongyloides* spp. peptidomics studies will rely on the continued expansion, improvement and integration of *in silico* datasets, peptidomics tools, LC–MS/MS technologies and optimized analysis pipelines. Specifically, future research in this area should prioritize the translation of the peptidomics pipelines used for *H. contortus* [150,151] and *C. elegans* [148] to probe *Strongyloides* spp., life cycle stage, somatic, ESP and EV peptidomes. These analyses will facilitate downstream functional analyses to inform aspects of *Strongyloides* peptide biology, including host–parasite–microbiome interactions.

## 7. *Strongyloides* small RNAs

sRNAs are non-coding RNA molecules of typically 18–30 nt in length that regulate genes at the post transcriptional level. The regulation of genes by sRNAs is an important mechanism required for the survival and reproduction of parasitic nematodes in the host and environment [161]. The role of sRNAs has been extensively studied in gene silencing, nematode development, transposon silencing and chromatin regulation [162]. sRNAs can be divided into three main classes: microRNAs (miRNAs), short-interfering RNAs (siRNAs) and PIWI-interacting RNAs (piRNAs). Microarrays, reverse transcription-quantitative PCR (RT-qPCR) and deep sRNA sequencing (sRNAseq) are commonly used for sRNA identification. Although both microarrays and RT-qPCR are valuable tools, these methods can only detect known and predefined sRNAs, and sRNAseq is the preferred method for identifying novel sRNAs.

sRNA sequencing has confirmed that sRNAs are expressed in *S. ratti*, *S. papillosus*, *S. stercoralis* and the close relative *P. trichosuri* [163–166]*.* The first study characterizing sRNA expression in *Strongyloides* was carried out in *S. ratti* iL3s and free-living adults [163]. Using a modified miRDeep2 pipeline [167], 106 mature and precursor *S. ratti* miRNA sequences were annotated, of which 18 were identified in mixed life cycle stages, eight in iL3s and 80 in both stages. Two miRNAs (*mir-34* and *mir-*71) were highlighted as potentially important in regulating stress and ageing. Further analysis on the miRNA seed sequence (the 2–8 nt region associated with regulating gene expression) revealed that 37 of the 106 miRNAs contain conserved miRNA seed families shared with *C. elegans* and *Pristionchus pacificus*, among 24 miRNA seed families conserved between the three species investigated [163]. sRNA expression in *S. ratti*, *S. papillosus* and *P. trichosuri* iL3s and free-living adults (mixed sex) was further compared using two different library preparations to capture sRNAs that have either a 5′ monophosphate or 5′ polyphosphate modification [164]. Annotation of *S. ratti* miRNAs identified 33 new miRNAs in addition to the 106 previously reported, bringing the total number of characterized miRNAs in *S. ratti* to 139 [164]. A total of 140 and 163 miRNAs have been annotated for *S. papillosus* and *P. trichosuri*, respectively, and miRNA sequences are largely conserved across *Strongyloides* spp. and *P. trichosuri*. miRNAs 22–23nt in length represent 79%, 55% and 80% of all genome-mapped sRNA reads in the 5′ monophosphate-enriched library for *S. ratti*, *S. papillosus* and *P. trichosuri*, respectively, and have a propensity for a 5′ uracil (U) in all three species [164]. Among all three species (*S. ratti*, *S. papillosus* and *P. trichosuri*), 65, 73 and 99 miRNAs, respectively, exhibited a differential level of expression between iL3s and free-living adult life cycle stages. *Strongyloides stercoralis* miRNA expression has been investigated in L1 and iL3s isolated from human stool [165]. A total of 385 and 208, mature and precursor miRNAs were identified in *S. stercoralis*, substantially more than the number of miRNAs identified in other *Strongyloides* spp. Upon comparison, 169 novel miRNAs showed no sequence similarity with miRNAs from *S. ratti*, *S. papillosus* or *C. elegans*, suggesting that there are many species-specific miRNAs in *S. stercoralis* or that miRNAs associated with the L1 stage in other *Strongyloides* spp. are yet to be discovered. Interestingly, the miRNAs expressed in iL3s were more transcriptionally active than those derived from L1s and were predicted to target and regulate the expression of parasitism-associated genes including astacin-like metalloproteases [165] (table 4).

**Table 4.** Summary of small RNA studies in *Strongyloides* species.

species	sRNA type	length of sRNA and 5′ nt	life cycle stage	modification	reference
*S. ratti*	miRNA	≥18 nt	iL3	5′ monophosphate	Ahmed *et al.* [163]
			free-living mixed stage		
*S. ratti*	miRNA	22–23U	iL3	5′ monophosphate	Holz & Streit [164]
*S. papillosus*			free-living mixed stage		
*P. trichosuri*					
*S. stercoralis*	miRNA	21–23U	L1	5′ monophosphate	Pomari *et al.* [165]
			iL3		
*S. ratti*	miRNA	21–23U	pF	5′ monophosphate	Suleiman *et al.* [166]
			free-living female		
*S. ratti*	siRNA	27GA	iL3	5′ all-phosphate	Holz & Streit [164]
*S. papillosus*			free-living mixed stage		
*P. trichosuri*					
*S. ratti*	siRNA	27GA	pF	5′ polyphosphate	Suleiman *et al.* [166]
			free-living female		
*S. ratti*	piRNA-like sRNA	21–22U	pF	5′ monophosphate	Suleiman *et al.* [166]

Non-miRNA classes of sRNAs including siRNAs and piRNAs have also been investigated in *Strongyloides*. *Strongyloides* spp. express sRNAs that originate from tRNAs, rRNAs, transposable elements (TEs) and intergenic sequences in the genome, hypothesized to be siRNAs [164]. These predicted siRNAs are more abundant in the 5′ modification-independent libraries, indicating that they have a 5′ modification such as a polyphosphate 5′ end, similar to the secondary siRNAs reported in *C. elegans*. The siRNAs expressed in *S. ratti*, *S. papilllosus* and *P. trichosuri* have a length of 27nt with an equal bias towards a 5′ guanine (G) and adenine (A) (27GAs). The targets of 27GAs based on sequence complementarity were predicted to be TEs in *S. ratti* pFs, free-living adults and iL3s, *S. papillosus* and *P. trichosuri* free-living adults and iL3s [164]. In *S. ratti* pF and flF, the 27GA siRNAs are predicted to target and regulate the expression of TEs predominantly located on the X-chromosome [166], but this information is not known for other species. A notable difference between 27GAs expressed in pF and flF life cycle stages is that the pF 27GAs mostly target DNA transposons, in comparison to the flF 27GAs that target retrotransposons [166]. Although sRNA data are available [165], siRNA expression in *S. stercoralis* has not yet been investigated.

The piRNA class of sRNAs has been lost in nematodes outside of clade V nematodes, including *Strongyloides* [68,161,164] evident from the absence of the Argonaute proteins from the PIWI family that interacts with and facilitates biogenesis of piRNAs. sRNA sequencing has not detected piRNA sequences in *S. ratti*, *S. papillosus* and *P. trichosuri* iL3 and free-living adults*.* However, the pF stage of *S. ratti* expresses a piRNA-like class of sRNAs with a length of 21–22 nt and propensity towards a 5′ uracil (21–22U) [166]. The piRNA-like sRNAs show striking resemblance to piRNAs of *C. elegans* and *D. melanogaster*, including their length, 5′ uracil, 5′ monophosphate modification, clustering and overlapping of sequences in the genome and an AT rich downstream sequence. However, these piRNA-like sRNAs did not have an upstream Ruby motif, as found for *C. elegans* piRNAs. The 21–22U piRNA-like sRNAs are specifically highly expressed in the pF compared with flF, indicating that they may be directly related to parasitism or features associated with the parasitic life cycle [166].

### (a) Comparison with *Caenorhabditis elegans*

sRNAs were originally identified in *C. elegans* and this species has become an important model organism for the study sRNA biology [168,169]. *C. elegans* expresses 253 precursors and 437 mature miRNAs [170]. These miRNAs have similar features to *Strongyloides* miRNAs, including a 5′ monophosphate modification, with a length of 21–22 nt and bias towards 5′ U or 5′ A. Comparison of *Strongyloides* miRNAs to those found in *C. elegans* has revealed that 31 seed families are shared between *S. ratti* and *C. elegans*, while 30 are shared between *S. papillosus* and *C. elegans* [164]. Among the three species, 29 seed families were shared that include miRNAs such as *let-7*, *lin-4*, *mir-34* and *mir-37*, indicating that the conservation of these miRNAs is essential in nematodes. In comparison to *Strongyloides*, *C. elegans* siRNAs and the sRNA pathways they belong to are well characterized, with distinct biological functions depending on their biogenesis. *C. elegans* expresses primary 26G siRNAs containing a 5′ monophosphate modification, which is important in the regulation of gene expression during spermatogenesis and zygotic development [171]. The 26G siRNAs, alongside piRNAs, can initiate the activation of secondary siRNAs, termed 22G siRNAs. The 22Gs contain a 5′ polyphosphate modification and have a role in regulating the expression of genes, pseudogenes and transposons, predominantly in the germline [172]. As there are no 26G and 22G siRNAs in *Strongyloides*, it has been suggested that 27GA siRNAs are equivalent to 22Gs in *C. elegans* [164,166]. As discussed above, unlike nematodes from clades I–IV—including *Strongyloides* spp.—*C. elegans* is known to produce and express piRNAs, also known as 21U sRNAs, that are important in regulating the activity of TEs [173]. *Strongyloides* have evolved an alternative class of piRNA-like sRNAs, the 21–22Us, which are predicted to be important in regulating TEs in the absence of piRNAs [166].

### (b) Comparison with other parasitic nematodess

Nematodes in clades I–V express conserved miRNAs [162]. However, similar to *Strongyloides*, piRNAs in clades I–IV nematode including *A. suum* (clade III) [174] and *B. pahangi* [175] are lost. Some of the species from clades III and IV have diverged and compensated for the loss of the PIWI pathway through higher expression of the secondary siRNA 22Gs that target and defend the germline against TEs, similar to the *C. elegans* piRNAs [161,174,176] and the piRNA-like sRNAs observed in *S. ratti* [166]. In addition to a role in regulating TEs and endogenous transcripts, sRNAs also have a role in parasite–host interactions. Parasitic nematodes secrete ELVs containing sRNAs that are internalized by host cells and alter host gene expression. The secretion of ELVs containing sRNAs was first identified in the parasitic nematode *H. polygyrus* [177]. These ELVs containing sRNAs suppress genes associated with innate immunity and are important for establishing parasitism in the host [178]. miRNAs in *H. polygyrus* ELVs shared sequence similarity with the host miRNAs. Further analysis of sRNAs using the 5′ phosphate-independent library revealed a prevalence of siRNAs derived from repetitive elements and intergenic regions [179].

### (c) Priority areas for future research

(i) sRNA expression across a range of *Strongyloide*s species. To gain a deeper understanding of the role of sRNAs in parasitism, it is crucial to conduct research on a range of life cycle stages, such as the pF and free-living adult stages of *S. stercoralis*, and species, such as *S. fuelleborni* and *S. venezuelensis*. (ii) Secretion of parasite-derived EVs containing sRNAs taken up by host cells. Further research is needed to understand the specific functions of sRNAs in these interactions, including how parasites use sRNAs to manipulate the host environment. This knowledge could not only enhance our understanding of host–parasite relationships, but also provide valuable diagnostic tools. ELV-bound sRNAs have not been investigated in *Strongyloides*. (iii) sRNAs as biomarkers. sRNAs have potential to be useful biomarkers for disease diagnosis and prognosis in humans and animals. As there is currently no gold standard method for detecting *Strongyloides* infections in the host, sRNAs present an opportunity to be developed as a diagnostic tool. For instance, the research carried out on sRNAs in *S. stercoralis* from human infections [165] provides insight into which sRNAs could be detected and exploited as putative biomarkers for the diagnostics of *Strongyloides* in humans. (iv) Validation of sRNAs and their targets. Most sRNA research to date has used sequencing followed by computational analysis, predicting targets through sequence complimentary. This work needs to be validated using high-throughput approaches such as Argonaute cross-linking immunoprecipitation (CLIP) [180] that identifies endogenous *in vivo* sRNA–target interactions, allowing us to better understand the role that sRNAs play in parasitism and host–parasite interactions. Understanding the regulatory networks of sRNAs could provide insight into their roles in the control of gene expression and other biological processes.

## 8. Transposable elements in *Strongyloides*

TEs are mobile genetic elements occupying the genomes of organisms across all branches of life. TEs can be a major driving force behind genetic variation but can also disrupt regular gene activity via different routes of mutagenesis [181]. The persistence of TEs in populations is maintained through vertical inheritance from one generation to the next. Although the study of TEs is not traditionally classified within the ‘omics’ field, unlike the large-scale study of genes, proteins and metabolites, recent advancements in sequencing technologies and downstream bioinformatic analyses have enabled researchers to explore TEs experimentally on a broader scale, akin to more conventional omics methodologies. Furthermore, omics methodologies such as transcriptomics and genomics enable comprehensive research into the TE biology of a species or at a population level, and can provide a deeper understanding of the effects of TEs on gene expression, genome organization and evolution. Eukaryotes have adopted post-transcriptional silencing via RNAi to counteract the harmful effects of TE insertions. The two known classes of sRNAs involved in these pathways in *Strongyloides* spp. are described above [164,166]. TEs are tightly controlled in the gonads of most animals because transposition into, and the potential disruption of sexual development genes can be lethal to the host organism [182]. In *S. ratti*, TE sequences are distributed throughout the genome but TE-associated sRNAs and their predicted TE targets are clustered on the X-chromosome of both *S. ratti* female adult stages [166].

Mutations induced by strong negative selection pressure have left TE sequences deteriorated and repetitive in nature, which makes their annotation notoriously difficult, especially in assemblies produced from short-read sequences. While several bioinformatics tools with *de novo* and homology-based algorithms have been developed to identify and annotate TEs, often a substantial amount of manual curation is still required to acquire a reliable TE consensus sequence library [183]. TE sequence annotation of the *S. ratti* genome has been carried out by four groups employing different methods of annotation: (i) Suleiman *et al*. [166] constructed a repeat library with RepeatModeler2 [184] and RepeatMasker [185] to annotate repeats via sequence comparison against the Dfam library [186]. As LTR retrotransposons seemed overrepresented in the *S. ratti* genome, they were further annotated by using the LTR specific tools LTRharvest [187] and LTRdigest [188]; (ii) WormBase ParaSite have recently announced a new repeat annotation feature, where RepeartModeler2 [184] was used to generate custom repeat models for all available genome assemblies including *S. ratti*, *S. venezuelensis*, *S. stercoralis* and *S. papillosus*, as well as close relatives *P. trichosuri* and *R*. *ditinus*. To annotate the repeat features, a pipeline including RepeatMasker [185], DustMasker [189] and TRF [190] was employed; (iii) Szitenberg *et al.* [191] have assembled TE libraries for several nematode species, including *S. ratti*, *S. venezuelensis*, *S. papillosus* and *P. trichosuri*. This curation used a homology-based method that based TE sequence searches on a nematode-specific *de novo* constructed library, rather than the more general Dfam or RepBase libraries [191,192]. After repeat sequence identification and compilation using RepeatModeler and RepeatMasker, a non-redundant library was pooled together using USEARCH [193] and One Code to Find Them All [194], while further consensus sequence classification was done with CENSOR [195] and LTRHarvest [187]. (iv) An *in silico* study conducted by the International Helminth Genomes Consortium constructed repeat libraries for the genomes of 56 parasitic and free-living nematodes. For each species, repeat libraries were constructed by combining libraries generated from RepeatModeler [184], TransposonPSI and LTRharvest [187]. The library was then used to mask genomic repeats using RepeatMasker [196].

**Figure 2.**
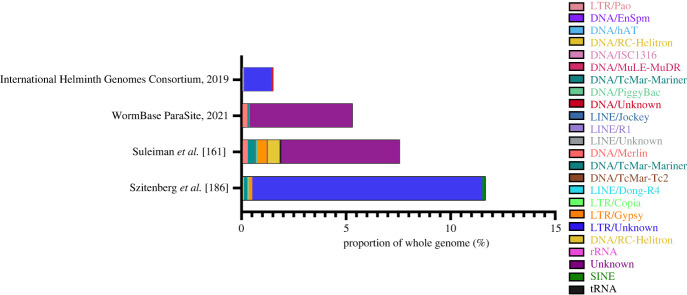
Comparison of TE consensus libraries for *S. ratti* for the four currently available TE libraries constructed by the International Helminth Genomics Consortium 2019 [179], WormBaseParaSite 2023 (v.18), Suleiman *et al.* [166] and Szitenberg *et al.* [191]. The percentage of the genome occupied by each TE family is illustrated on the *x*-axis and annotation of different classes of TE are highlighted by colour. (Online version in colour.)

The curation of TE annotations for *Strongyloides* spp. by Suleiman *et al.* [166] and WormBase ParaSite have run RepeatMasker [185] with its native Dfam and Repbase libraries that consist of eukaryotic repeat sequences predominantly belonging to model organisms. Hence, repeat sequences and TEs belonging exclusively to non-model organisms would not be as accurately represented. Conversely, the Szitenberg and International Helminth Genomes Consortium consensus libraries for *S. ratti*, *S. venezuelensis*, *S. papillosus* and *P. trichosuri* [191] have been built by running RepeatMasker with custom de novo nematode libraries based on repeat sequences generated through running RepeatModeler in addition to other tools mentioned above. This technique would offer a more specific annotation through the identification of species-specific repeats that would not be as represented by using the more universal Dfam and RepBase libraries on their own. The Szitenberg *S. ratti* TE library comprises 657 TE families occupying 11.2% of the genome, while the International Helminth Genomes Consortium library consists of 3 TE families covering 1.8% of the genome. The Suleiman library contains 5526 TE families that comprise 8.45% of the genome, and the WormBase ParaSite library contains 111 TE families that cover 5.64% of the genome. In addition to mapping to variable proportions of the *S. ratti* genome, the four TE consensus libraries also vary in the annotation and quantification of different TE families (figure 2). An unknown TE class occupies much of the genome according to the WormBase ParaSite and Suleiman libraries. The DNA element Merlin is also shown to occupy approximately 0.25% of the genome by both libraries. An LTR belonging to an unknown family is shown to have the highest level of expansion in the *S. ratti* genome by the Szitenberg and the International Helminth Genomes Consortium annotations. Interestingly, this expansion has not been highlighted in the WormBase ParaSite and Suleiman annotations, and instead an unknown TE has shown increased genomic proliferation. The unknown TE family emphasized in the WormBase ParaSite and Suleiman libraries could in fact be the unclassified LTR family highlighted in the Szitenberg and the International Helminth Genomes Consortium libraries. This element could have been highlighted in only two of the available *S. ratti* repeat annotations due to the use of de novo libraries during the analysis or as a result of different cut-offs and LTR characterization techniques. The DNA transposon TcMar-Mariner and the LTR Gypsy elements are highlighted in the genome by all libraries at different amounts apart from the International Helminth Genomes Consortium library. The International Helminth Genomes Consortium annotation covers the lowest proportion of the *S. ratti* genome as their annotation only represents specific TE families. RC-Helitron DNA elements are annotated in the Szitenberg and Suleiman libraries, where it is shown at a higher abundance by the Suleiman library. SINEs have only been annotated and detected by the Szitenberg TE annotation.

### (a) Comparison with *Caenorhabditis elegans*

Despite being the first animal to have its genome sequenced and assembled to a complete level, little is known about TE dynamics in the model organism *C. elegans.* Various methods have been used to annotate the repetitive sequences, including TEs, in the *C. elegans* genome [197–200] and consistently conclude that TEs occupy approximately 12% of the genome. Similar to *Meloidogyne* spp. genomes, DNA transposons make up the majority of expanded TEs. Furthermore, repetitive sequences of the free-living nematode *P. pacificus* were recently annotated via a custom ‘sliding window’ method, where 1 kbp sequence ‘windows’ are extracted and analysed for repeats using 11 different *de novo* and library-based tools illustrating that 24% of the *P. pacificus* genome consists of TEs [201]. Similar to the genome of *S. ratti*, retrotransposons are the most abundant class of TEs in the *P. pacificus* genome, accounting for 50% of masked repeats.

### (b) Comparison with other parasitic nematodes

The International Helminth Genomes Consortium TE annotation of 56 helminth genomes was analysed via a multiple regression model to reveal that variations in genome size within the nematode phylum were primarily influenced by variations in genome repeat content, ranging from 3.8% to 54.5%. Notably, the significant contributors to this variation were LTRs, DNA transposons and simple repeats [196]. The aforementioned libraries were constructed from genome assemblies produced by short-read sequences, which are limited in their ability to fully represent TE content due to the repetitive and divergent nature of these elements' sequences [202]. Combining long-read and short-read sequences can increase assembly accuracy and aid in improving the annotation of repetitive regions and TE boundaries [203]. The genome of the plant parasite *Meloidogyne enterolobii* was recently sequenced and assembled using PacBio and Illumina reads [204]. A TE consensus library was built by utilizing the Tedenovo pipeline [205] for TE predictions. The clustering of similar sequences was carried out using Recon [206], where a multi-sequence alignment of similar clusters was then completed to deduce a consensus sequence using MAP [56] and PASTEClassifier [207]. It was found that 47.62% of the *M. enterolobii* genome is composed of repetitive elements. However, only 8.7% of the repeats were annotated as TEs, where DNA elements seem to have expanded at a greater rate (5.83%) than retrotransposons (2.87%). A consensus TE library was deduced for the closely related species *M. incognita* using the same workflow described by Koutsovoulos *et al*. [204]. The repeatome was shown to span 26.38% of the genome, 4.67% of which is subjugated by retrotransposons (0.9%) and DNA elements (3.77%) [208]. *Meloidogyne* spp. have a lower proportion of TEs in their genomes compared to *Strongyloides* spp. despite them leading similar parasitic lifestyles. Furthermore, DNA transposons have experienced a much larger expansion in the genomes of *Meloidogyne* spp. in comparison to *S. ratti*, whose genome seems to include a large number of LTRs. The repeatome of the pig parasite *A. suum* was also identified using a combination of homology and *de novo*-based tools [176]. The *Ascaris* genome was assembled using long PacBio reads, revealing that approximately 41% of the genome comprises repetitive sequences. However, the specific classification of these repeats and the familial identities of *Ascaris* TEs have not been reported [171].

### (c) Areas for future priority

TEs contribute to gene regulation by acting as regulatory elements that manipulate transcription and epigenetic states [209]. By studying TE activity in parasitic and free-living stages of *Strongyloides* spp., researchers have the opportunity to explore potential variations in transposition and TE repression patterns between free-living and parasitic stages. This exploration may elucidate the molecular mechanisms responsible for variances in gene regulation and epigenetic patterns resulting from TE activity, particularly within germline cells [210] and how these mechanisms relate to different lifestyles, i.e. parasitic versus free-living, parthenogenic versus sexual, long versus short lifespans (pF versus flM/flF, respectively). To better understand *Strongyloides* TEs, a library consisting of consensus sequences corresponding to the different TE families present in the genomes of *Strongyloides* species must be curated. Utilizing long and short sequencing in a *de novo* pipeline as described by Koutsovoulos *et al.* [204] could be used for *Strongyloides* spp*.* to construct consensus libraries. However, as some previously uncharacterized TE families could be *Strongyloides*-specific, some manual curation will be required to compile an accurate consensus library, as described by Goubert *et al.* [183]. The established consensus library can be employed in transcriptomic analyses to examine TE dynamics across life cycle stages. Additionally, exploring different histone marks in the context of masked TEs within the *Strongyloides* genome will allow for a deeper investigation into the role of TEs in genome architecture and their expression patterns. On a broader scale, accurately annotated TE libraries can facilitate comparative studies on TE load across different species and populations. These results can shed light on whether factors such as environment, lifestyle and stress influence the abundance and diversity of an organism's TE dynamics, and to help elucidate what regulatory roles differences in composition, load and activity may play. Furthermore, investigating the TE content of diverse *Strongyloides* species and strains inhabiting different environments can shed light on the impact of the environment on genome organization, as shown previously in the parasitic flatworms *Schistosoma mansoni* and *S. japonicum* [211].

## 9. Conclusion

Recent advances in technologies and omics analysis pipelines will continue to reduce the gap between omics data availability for *Strongyloides* spp. and *C. elegans*. Moving forward, the priority in *Strongyloides* omics includes (i) investigating more species, including those important to human and livestock health; (ii) investigating samples from wild and clinically important settings as well as laboratory-based populations to understand variation and diversity; and (iii) the application of the latest technologies e.g. long-read sequencing, to improve genome and transcriptome assemblies. There are also areas of omics that have received little or no attention, such as peptidomics, metabolomics, long non-coding RNA, chromatin organization and histone modifications. Numerous projects are now underway within the *Strongyloides* scientific community to address these areas, which will further close the gap between *C. elegans* and *Strongyloides* spp. and improve our understating of the omics biology underpinning parasitism. Advances in omics technologies and data availability will aid identification and development of new ways to detect and control *Strongyloides* infections and treat the disease that they cause.

## Data Availability

This article has no additional data.
